# Electron interaction with copper(II) carboxylate compounds

**DOI:** 10.3762/bjnano.9.38

**Published:** 2018-02-01

**Authors:** Michal Lacko, Peter Papp, Iwona B Szymańska, Edward Szłyk, Štefan Matejčík

**Affiliations:** 1 Department of Experimental Physics, Faculty of Mathematics, Physics and Informatics, Comenius University in Bratislava, Mlynská dolina F2, 842 48 Bratislava, Slovakia; 2 Faculty of Chemistry, Nicolaus Copernicus University in Toruń, Gagarina 7, 87-100 Toruń, Poland

**Keywords:** amines, dissociative electron attachment, dissociative ionization, FEBID, low energy electrons interaction

## Abstract

In the present study we have performed electron collision experiments with copper carboxylate complexes: [Cu_2_(*t*-BuNH_2_)_2_(µ-O_2_CC_2_F_5_)_4_], [Cu_2_(*s*-BuNH_2_)_2_(µ-O_2_CC_2_F_5_)_4_], [Cu_2_(EtNH_2_)_2_(µ-O_2_CC_2_F_5_)_4_], and [Cu_2_(µ-O_2_CC_2_F_5_)_4_]. Mass spectrometry was used to identify the fragmentation pattern of the coordination compounds produced in crossed electron – molecular beam experiments and to measure the dependence of ion yields of positive and negative ions on the electron energy. The dissociation pattern of positive ions contains a sequential loss of both the carboxylate ligands and/or the amine ligands from the complexes. Moreover, the fragmentation of the ligands themselves is visible in the mass spectrum below *m*/*z* 140. For the studied complexes the metallated ions containing both ligands, e.g., Cu_2_(O_2_CC_2_F_5_)(RNH_2_)^+^, Cu_2_(O_2_CC_2_F_5_)_3_(RNH_2_)_2_^+^ confirm the evaporation of whole complex molecules. A significant production of Cu^+^ ion was observed only for [Cu_2_(µ-O_2_CC_2_F_5_)_4_], a weak yield was detected for [Cu_2_(EtNH_2_)_2_(µ-O_2_CC_2_F_5_)_4_] as well. The dissociative electron attachment processes leading to formation of negative ions are similar for all investigated molecules as the highest unoccupied molecular orbital of the studied complexes has Cu–N and Cu–O antibonding character. For all complexes, formation of the Cu_2_(O_2_CC_2_F_5_)_4_^−•^ anion is observed together with mononuclear DEA fragments Cu(O_2_CC_2_F_5_)_3_^−^, Cu(O_2_CC_2_F_5_)_2_^−^ and Cu(O_2_CC_2_F_5_)^−•^. All dominant DEA fragments of these complexes are formed through single particle resonant processes close to 0 eV.

## Introduction

Present technological changes require the development of new methods and new materials for preparation of thin layers or 3D nanostructures. Complexes and metalorganic compounds are used as precursors in modern nano scale layer techniques. After activation, molecules undergo dissociation on the surface. Volatile parts of molecules are removed from the surface while a metal component remains and forms the layer. Activation of the precursor molecules can be induced by several processes. For instance, a catalytic or a thermal dissociation can occur. Plasma activated processes such as plasma enhanced chemical vapor deposition (PECVD) can be used for coating of the surface [1]. In the latter, reactive chemical species (radicals) and electrons lead to activation of molecules and this process can be controlled well on large scales.

One of the most innovative techniques, known as EBID or FEBID (Focused Electron Beam Induced Deposition) [2–3], uses a high energy electron beam that can be focused into a spot of diameter in the nanometer range for a spatially confined activation of precursor molecules on a very narrow range of the surface. The presence of high energy electrons from the primary beam (usually around 10 keV) causes ionization inside the wafer, with a high yield of secondary low energy electrons (below 100 eV). These electrons can diffuse to the surface and initiate reactions in the precursor molecules. As a result, a deposit is formed. This technique enables the production of free standing 3D nanostructures and is already used commercially for the repair of photolithographic masks [4–5].

However, the underlying chemical reactions on the surface are still not well known. Moreover, the main problems of FEBID are co-deposited impurities resulting from incomplete dissociation of the precursor molecules. The level of purity strongly depends on the type of the precursor molecule. Only a few types of precursors are known to produce a layer with purity over 80% [3]. Moreover, there is no clear connection between the layer purity and the type of ligand in the precursor; an iron deposit from Fe(CO)_5_ leads to purity over 95% of Fe, while tungsten layers from W(CO)_6_ can reach purities of W from 55 to 70% [6–7]. For comparison, the deposition of cobalt from Co(CO)_3_(NO) leads to around 50% purity [8] or satisfying purity over 95% using the dimer Co_2_(CO)_8_ [9]. Only few types of precursor molecules can be converted into a layer with satisfying level of purity, for other elements there is still demand for new precursors with other satisfactory parameters like vapor pressure, toxicity, thermal stability, and stability over time.

Electrons present on the surface are combination of high energy electrons from the primary electron beam and secondary electrons emitted from the surface. High energy electrons have low interaction cross section with the target molecule; their interaction efficiency is therefore very low. Secondary electrons, on the other hand, play important role through electron attachment (EA), dissociative electron attachment (DEA) [10–16] and electron ionization (EI), dissociative ionization (DI) [17–19] processes. Their kinetic energy is only a few eV, with energy distribution determined by the type of wafer and energy of primary beam [20–21]. Thorman et al. have compared gas phase and surface data on low energy electron interactions with some commonly used FEBID precursors [22] and have shown that in some cases a single ligand loss dominates the initial fragmentation following electron induced ionization or attachment. This may then induce other surface interactions. They also conclude that dissociation through neutral dissociations induced via electron impact excitation [23] can be as important as DEA.

Copper is an important material commonly used in the advanced metallization of microelectronic and optoelectronic devices and ultralarge-scale integrated (ULSI) circuits due to its low electrical resistivity, high stress-induced deformation, and electromigration resistance which is higher than for aluminum [24–27]. Copper nanostructures, especially nanowires, are applied in opto-electronic devices, solar cells, field-emission displays, catalysis, electronic skins, and sensor devices. Moreover, they can find medical applications because copper exhibits antibacterial and antifungal properties [28].

In FEBID experiments, the deposited Cu–C lines and squares, obtained from the fluorinated copper(II) β-diketonate [Cu(hfac)_2_], had an atomic ratio of approximately **Cu**/C/O/F = **10**:64:25:1. In materials obtained using other copper(I) β-diketonate complexes, namely, [Cu(hfac)VTMS], [Cu(hfac)DMB], and [Cu(hfac)MHY] the atomic ratios of **Cu**/C/O/Si were **20–45**:35–70:8–14:2–10, **25–60**:15–60:5–25:-, and **13**:82:3:-, respectively. As-deposited freestanding rods from FEBID experiments with [Cu(hfac)VTMS] showed small Cu nanocrystals dispersed in a polymeric carbonaceous matrix, which contains all the ligand elements: carbon, oxygen, fluorine and silicone [29–30].

Therefore, new copper FEBID precursors are still necessary. When designing such new precursors, it should be considered that copper(II) derivatives are more user-friendly than copper(I) compounds, which are usually air and moisture sensitive, which results in decomposition of the precursor itself. Also, introduction of amine ligands was expected to be advantageous. In fact, the reducing action of ammine ligands was discussed previously with respect to FEBID experiments using cisplatin [Pt(NH_3_)_2_Cl_2_] as precursor [31]. These latter experiments were motivated by gas phase DEA studies on this compound showing also that it can be evaporated intact [32].

Electron impact MS spectra analysis of [Cu(O_2_CR)_2_] (R = C*_n_*H_2_*_n_*_−1_, *n* = 1–3; CF_3_, CHF_2_, C_6_H_5_, p-F-C_6_H_4_, p-Cl-C_6_H_4_, o-Cl-C_6_H_4_, C_6_F_5_) [33] and [Cu_2_(*t*-BuNH_2_)_2_(µ-O_2_CR)_4_] (R = C*_n_*F_2_*_n_*_−1_, *n* = 1–6) [34] investigated previously suggested that copper(II) easily changes its oxidation state to Cu(I) in the gas phase and the highest intensity was observed for the fragment Cu_2_(O_2_CR)^+^. On the other hand, copper(I) compounds spontaneously disproportionate to copper(II) derivatives and copper(0), which can form the deposit.

Gas phase electron–precursor collision experiments should allow to determine the potential usefulness of new compounds in FEBID. Moreover, these results can be interesting for the development of new metalorganic or coordination compounds suitable for FEBID.

Previous gas phase studies performed in Bratislava with coordination compounds have shown the importance of DI and DEA processes in FEBID [10–12,17–19,35]. The partial decomposition of the metal complex via DI and DEA together with the role of secondary electrons in FEBID acting on much wider range than is the focus of the primary beam leads to broadening of the deposited structure [36]. The importance of the DI and/or DEA processes in FEBID was also discussed by Warneke et al. [37] with the focus on the appropriate choice of the ligands on the metal complex. In this work, acetylacetone was studied under electron impact and it was concluded from gas phase experiments that radicals released by electron-induced fragmentation react with intact molecules to produce a non-volatile residue that was detected using XPS. These results emphasize that both the proper choice of ligands and the knowledge of elementary processes under DI and DEA in gas phase are important for the understanding and development of FEBID precursors.

**Figure 1:**
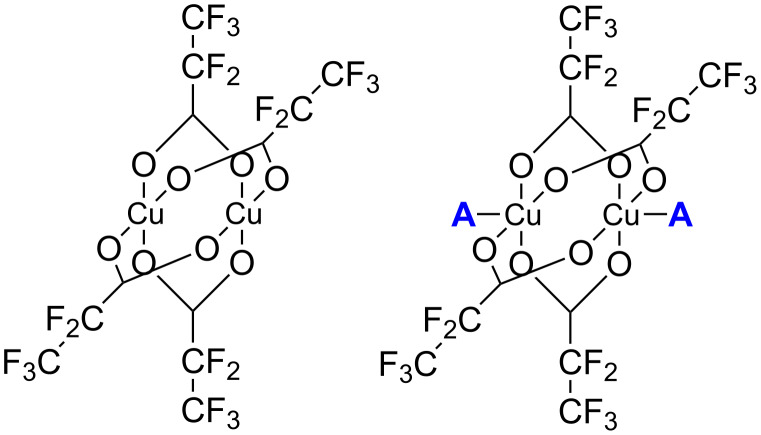
A schematic structure of investigated molecules (left) [Cu_2_(µ-O_2_CC_2_F_5_)_4_], (right) [Cu_2_(EtNH_2_)_2_(µ-O_2_CC_2_F_5_)_4_], [Cu_2_(*t*-BuNH_2_)_2_(µ-O_2_CC_2_F_5_)_4_], and Cu_2_(*s*-BuNH_2_)_2_(µ-O_2_CC_2_F_5_)_4_], where A represent different amine ligands.

In the present experiments electron impact ionization, electron attachment, and subsequent dissociation processes have been studied for the first time on the following copper(II) pentafluoropropionate derivatives: [Cu_2_(*t*-BuNH_2_)_2_(µ-O_2_CC_2_F_5_)_4_], [Cu_2_(*s*-BuNH_2_)_2_(µ-O_2_CC_2_F_5_)_4_], [Cu_2_(EtNH_2_)_2_(µ-O_2_CC_2_F_5_)_4_], and [Cu_2_(µ-O_2_CC_2_F_5_)_4_] (Figure 1).

The carboxylate copper(II) complexes with *tert*-butylamine ([Cu_2_(*t*-BuNH_2_)_2_(µ-O_2_CR)_4_], where R = C*_n_*F_2_*_n_*_+1_, *n* = 1–6) were previously applied as Cu CVD precursors [34,38] for the formation of copper nanomaterials. This fact confirms that copper(II) carboxylate compounds can be considered as copper sources in vapor deposition processes. The influence of secondary ligands on the physicochemical properties and, with regards to FEBID, the electron-induced fragmentation behaviour of pentafluoropropionate Cu(II) complexes as studied here is thus of particular interest.

## Results

### Mass spectrum of [Cu_2_(µ-O_2_CC_2_F_5_)_4_]

**Figure 2:**
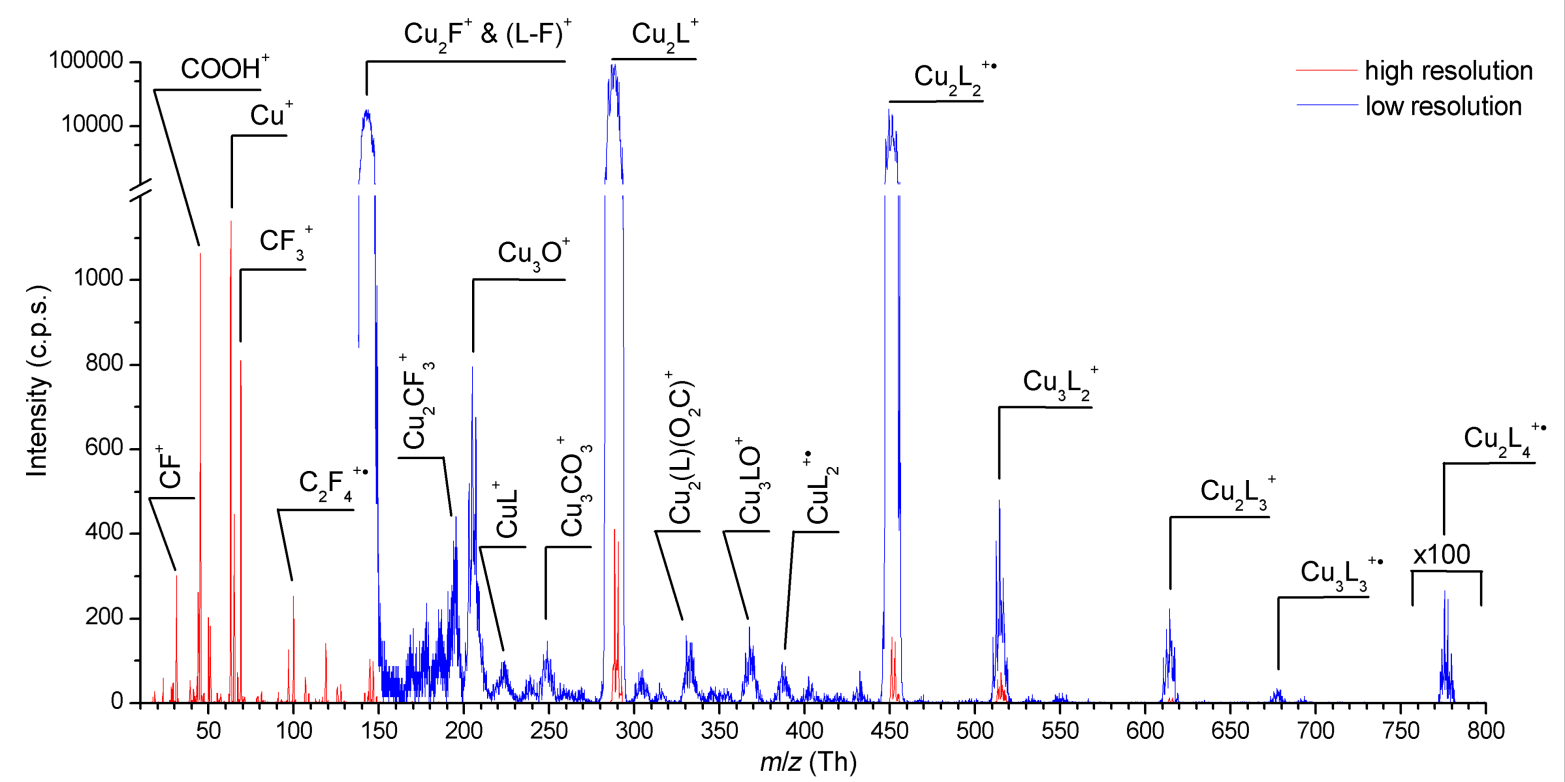
Mass spectrum of [Cu_2_(µ-O_2_CC_2_F_5_)_4_] molecule over the range *m*/*z* 10–800 obtained at electron energy 70 eV and temperature of the beam source of ≈150 °C. The notation L = O_2_CC_2_F_5_ was used in the mass spectrum. The spectral range below *m*/*z* 150 and selected peaks at higher masses were measured with higher mass resolution (red line) while the range above *m*/*z* 150 was registered with lower resolution (blue line) to increase the signal.

[Cu_2_(µ-O_2_CC_2_F_5_)_4_] represents the basic chemical structure for all discussed carboxylate compounds in this work. In Figure 2 the fragmentation pattern of [Cu_2_(µ-O_2_CC_2_F_5_)_4_] is presented. The spectrum was obtained with higher mass resolution at which it is easy to resolve to atomic masses for *m*/*z* from 10 to 150. For *m*/*z* from 150 up to 800 lower mass resolution was used to increase the signal intensity. Some of the peaks obtained at low resolution but with satisfying intensity were re-measured with higher resolution again.

The parent ion Cu_2_(µ-O_2_CC_2_F_5_)_4_^+•^ has been detected at *m*/*z* 788. Its electron induced dissociation can be characterized by several fragmentation patterns. The first is the sequential loss of the ligand radical O_2_C–C_2_F_5_ (L), which is characterized by formation of Cu_2_L_3_^+^, Cu_2_L_2_^+•^, and Cu_2_L^+^ ions up to a final decomposition to the copper dinuclear Cu_2_^+•^ and the Cu^+^ ion as well. Another pattern results from the fragmentation of the O_2_C–C_2_F_5_ ligand itself, as can be seen for the C–C bond dissociation between the CO_2_ and C_2_F_5_ units. The rupture of this bond in the coordinated carboxylate gives rise to the Cu_2_(L)(O_2_C)^+^ ion. The remaining products are formed via additional fragmentation of the ligand or rearrangement of some of its atoms, e.g., Cu_2_CF_3_^+^. Moreover, we have identified the simultaneous C–F and C–C bond dissociation via fragmentation of neutral tetrafluoroethene (C_2_F_4_) from the complex, while the remaining fluorine atom is bound in Cu_2_F^+^. Finally, the formation of the Cu^+^ ion has been observed only in the mass spectrum at higher temperature (150 °C) of the molecular beam source. This copper fragment abundance achieved the highest value among signals registered below *m*/*z* 300. Over this *m*/*z* range the signal intensity is not reduced by the QMS (Experimental).

Additionally, we have seen tricopper Cu_3_LO^+^ (*m*/*z* 368) and Cu_3_O^+^ (*m*/*z* 205) ions. The formation of tricopper ionic fragments could be a consequence of structural units containing three Cu atoms in the solid phase of [Cu_2_(µ-O_2_CR)_4_]. These can be formed via a possible interaction of oxygen atoms and copper atoms of two neighbouring dinuclear structures. The X-ray crystal structure of studied compound is still unresolved but the trinuclear (or more complicated) structures for copper(II) carboxylates were observed [39–41].

The presence of a ligand cation O_2_CC_2_F_5_^+^ (L^+^) has not been confirmed in the positive ion mass spectrum of [Cu_2_(µ-O_2_CC_2_F_5_)_4_], in contrast to the DEA spectra (see below) where L^−^ was formed. In the [Cu_2_(µ-O_2_CC_2_F_5_)_4_] mass spectrum the formation of the pentafluoroethyl ion (C_2_F_5_^+^) was observed, which further dissociated to C_2_F_4_^+•^, CF_3_^+^, CF_2_^+^, and CF^+^ ions. The complementary reaction is the formation of the CO_2_^+•^ cation.

An unusual product in the mass spectrum of this molecule can be seen at *m*/*z* 45, which is according to our conclusions the COOH^+^ ion. The only explanation for the formation of this product from a sample without any hydrogen atom is the interaction between [Cu_2_(µ-O_2_CC_2_F_5_)_4_] and adsorbed water traces. The possibility that the [Cu_2_(µ-O_2_CC_2_F_5_)_4_] sample was contaminated by some free acid was excluded.

**Figure 3:**
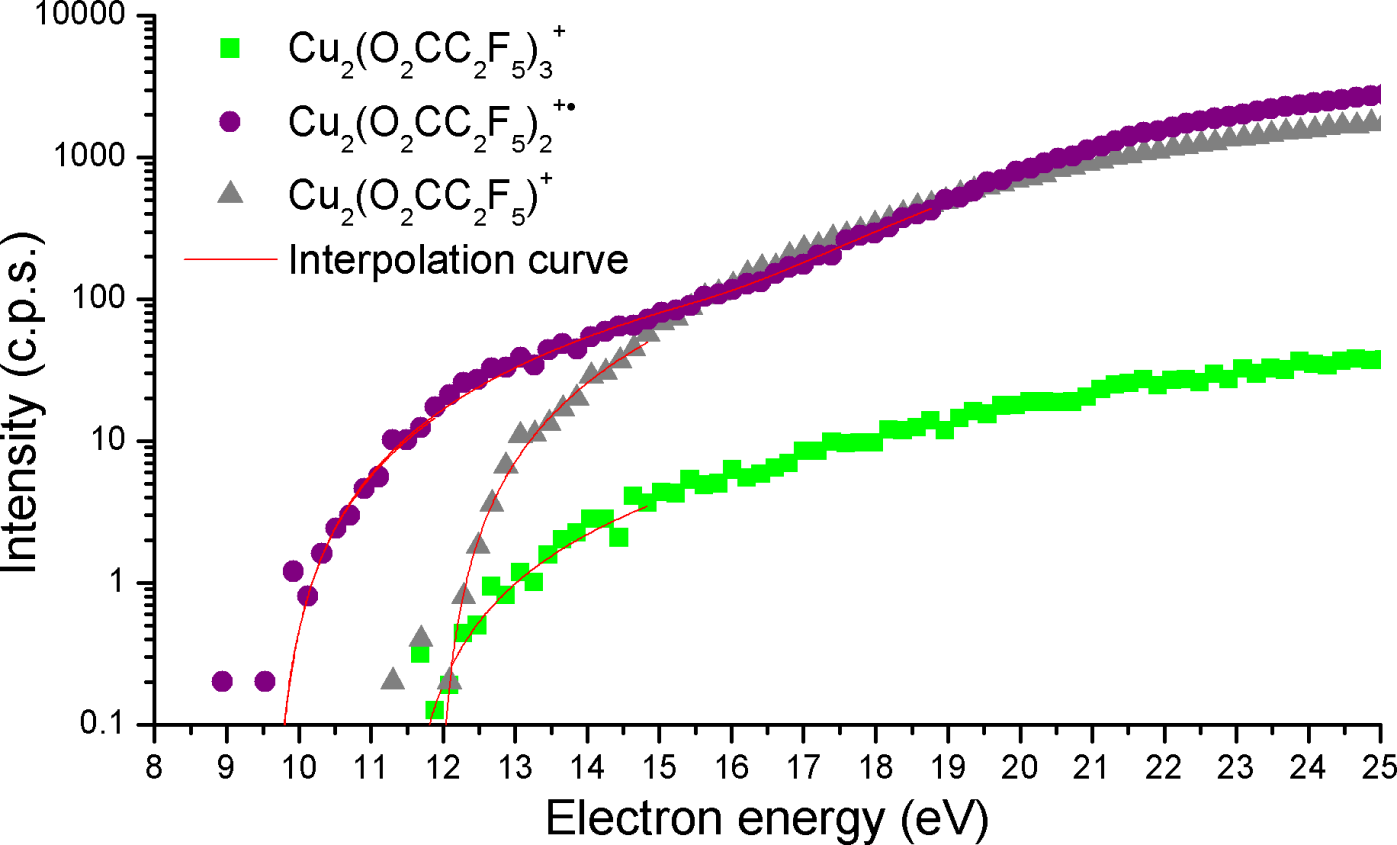
Relative cross sections of the dissociative ionization of the [Cu_2_(µ-O_2_CC_2_F_5_)_4_] molecule as a result of a ligand loss fragmentation process. Red lines represent a theoretical fits using the modified Wannier formula.

Appearance energies have been measured for several fragments (Figure 3). The values presented in Table 1 are estimated with uncertainty ±0.5 eV, which results from the resolution of the electron beam that varied from 100 meV up to 500 meV. The largest value was taken to estimate the margin of error. The resolution of the electron beam was lowered to increase the electron current and ion yields when the sample has adsorbed on the electrodes of the monochromator and thus affected their electric fields. The appearance energy for the ion with *m*/*z* 18 is *AE*_18_ = 12.6 eV and is in a good agreement with the ionization energy of the water molecule, *IE*(H_2_O) = 12.62 eV [42]. It cannot be produced from [Cu_2_(µ-O_2_CC_2_F_5_)_4_] itself due to its lack of hydrogen atoms; obviously must have desorbed from the sample. This is in agreement with the presence of the previously discussed COOH^+^ ion that is assumed to be formed via interaction of residual water molecules with the sample.

**Table 1:** Appearance energies for selected ions from dissociation of [Cu_2_(µ-O_2_CC_2_F_5_)_4_] induced by electron impact ionization.

*m*/*z*	Ion	*AE*^1^[eV]	*AE*^2^[eV]	*m*/*z*	Ion	*AE*^1^[eV]	*AE*^2^[eV]

18	H_2_O^+•^	12.6^a^		145	Cu_2_F^+^	15.9^a^	
31	CF^+^	11.0^a^	18.3^b^	289	Cu_2_L^+^	11.9^a^	
45	COOH^+^	13.5^a^		370	Cu_3_LO^+^	25.7^c^	
50	CF_2_^+^	14.4^a^		452	Cu_2_L_2_^+•^	09.7^a^	15.2^b^
63	Cu^+^	15.7^a^		515	Cu_3_L_2_^+^	16.1^a^	
69	CF_3_^+^	11.7^a^	16.1^a^	615	Cu_2_L_3_^+^	11.4^a^	15.7^b^
100	C_2_F_4_^+•^	12.2^a^		678	Cu_3_L_3_^+•^	15.7^a^	
119	C_2_F_5_^+^	15.1^a^					

^a^Uncertainty ±0.5 eV, ^b^uncertainty ±1 eV, ^c^uncertainty ±2 eV.

Dissociation of first carboxylate ligand from [Cu_2_(µ-O_2_CC_2_F_5_)_4_] molecule was detected with *AE*^1^_615_ = 11.4 eV and with a second threshold at *AE*^2^_615_ = 15.7 eV. Dissociation of second ligand occurs with *AE*^1^_452_ = 9.7 eV and again with a second threshold at *AE*^2^_452_ = 15.2 eV. Dissociation of third ligand starts at *AE*_289_ = 11.9 eV without any other distinguishable threshold. The origin of the second threshold can be a) an energetically higher excited state of the same fragment, b) a different stoichiometric fragment or c) a different energetically higher process, or doubly charged product. We did not find any other stoichiometric product for the given masses so the existence of an excited ionic state is the reasonable explanation. An interesting fact is that the loss of two ligands requires less energy than the dissociation of first and third ligand.

As mentioned before we did not observe any signal for a ligand cation itself, therefore we did not obtain any *AE* value for its production. A decreasing trend of *AE* for C_x_F_y_ fragments was found: *AE*_119_ = 15.1 eV, *AE*_100_ = 12.2 eV, *AE*_69_ = 11.7 eV, *AE*_31_ = 11.0 eV except of CF_2_^+^ ion (*m*/*z* 50) that clearly requires additional energy for dissociation of F atom from CF_3_^+^ ion. Ion COOH^+^ with *m*/*z* 45 proposed as an impurity has *AE*_45_ = 13.5 eV, which is however much higher than the ionization energy of hydrocarboxyl radical *IE*(COOH) = 8.2 eV [43]. According to our conclusions its formation is accompanied by dissociation of two Cu–O bonds and a C–C bond on the complex as well as O–H bond dissociation on the water molecule.

### Mass spectra of [Cu_2_(RNH_2_)_2_(µ-O_2_CC_2_F_5_)_4_] complexes

**Figure 4:**
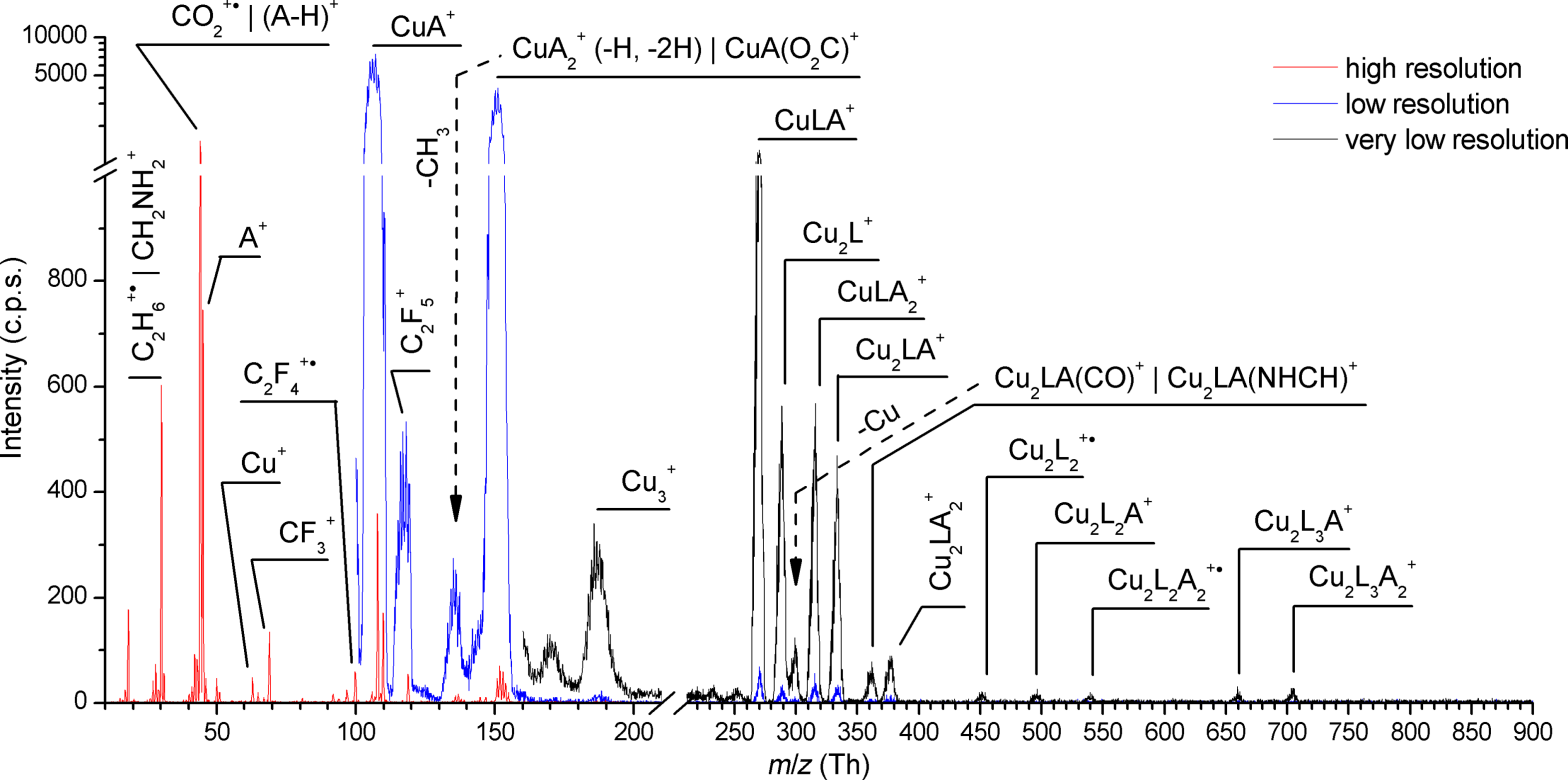
Mass spectrum of [Cu_2_(EtNH_2_)_2_(µ-O_2_CC_2_F_5_)_4_] molecule over the range *m*/*z* 10–900 obtained at electron energy 70 eV and temperature of the beam source of ≈80 °C. The spectral range below *m*/*z* 200 was measured with higher mass resolution (red line), while that range above *m*/*z* 100 (blue line) and above *m*/*z* 160 (black line) was measured with lower mass resolutions to increase the signal.

Compared to [Cu_2_(µ-O_2_CC_2_F_5_)_4_], the fragmentation pattern of [Cu_2_(EtNH_2_)_2_(µ-O_2_CC_2_F_5_)_4_] (Figure 4) is strongly affected by the presence of two ethylamine ligands and contains a combination of losses of both types of ligands. The parent ion was not detected and the highest mass signal (*m*/*z* 704) for the Cu_2_L_3_A_2_^+^ (A – amine ligand) fragment was observed. The detachment of the first, second as well as third carboxylate ligand is usually accompanied by the loss of the ethylamine ligand forming Cu_2_L_x_A^+^. The loss of the second ethylamine is present only for dissociation of two (Cu_2_L_2_^+•^ *m*/*z* 452) or three (Cu_2_L^+^ *m*/*z* 289) carboxylate ligands from the complex but not for one only. Finally, dissociation of all carboxylate ligands has been observed with the dicopper system disconnection forming the CuA_2_^+^ and CuA^+^ ions (*m*/*z* 153 and 108). As in the case of [Cu_2_(µ-O_2_CC_2_F_5_)_4_] discussed before, the fragmentation of the coordinated ligands themselves has been observed creating, e.g., Cu_2_LA(CO)^+^/Cu_2_LA(NH_2_CH)^+^, CuA(O_2_C)^+^. The Cu_3_^+^ fragment is also detected. We propose that the [Cu_2_(EtNH_2_)_2_(µ-O_2_CC_2_F_5_)_4_] complex partly loses the amine ligand (A) when it is heated in vacuum chamber and forms the structure similar to [Cu_2_(µ-O_2_CC_2_F_5_)_4_]. The formation of tricopper complex via copper oxygen interaction of two neighboring molecules can occur as was already discussed above for the [Cu_2_(µ-O_2_CC_2_F_5_)_4_] compound.

The dissociation of the carboxylate ligand is again visible in the mass spectrum, but not dominant anymore. Series of peaks at *m*/*z* 30 and *m*/*z* 45 uncover the preferable dissociation through ethylamine ligand fragmentation. A peak at *m*/*z* 44 can be assigned to CO_2_^+•^ or the (A-H)^+^ ion. The abundance of the CO_2_^+•^ ion released from described above [Cu_2_(µ-O_2_CC_2_F_5_)_4_] is comparable with other fragments of carboxylate ligand. However, in the case of [Cu_2_(EtNH_2_)_2_(µ-O_2_CC_2_F_5_)_4_], the abundances of amine fragments (e.g., C_2_H_6_^+•^/CH_2_NH_2_^+^) were changing in the same manner as for *m*/*z* 44 and in opposite to the fluorine-containing fragments of the carboxylate ligand. These facts suggest that *m*/*z* 44 relates to the formation of (A-H)^+^. Moreover, in comparison with [Cu_2_(µ-O_2_CC_2_F_5_)_4_], the creation of the pure copper ion is strongly suppressed.

**Figure 5:**
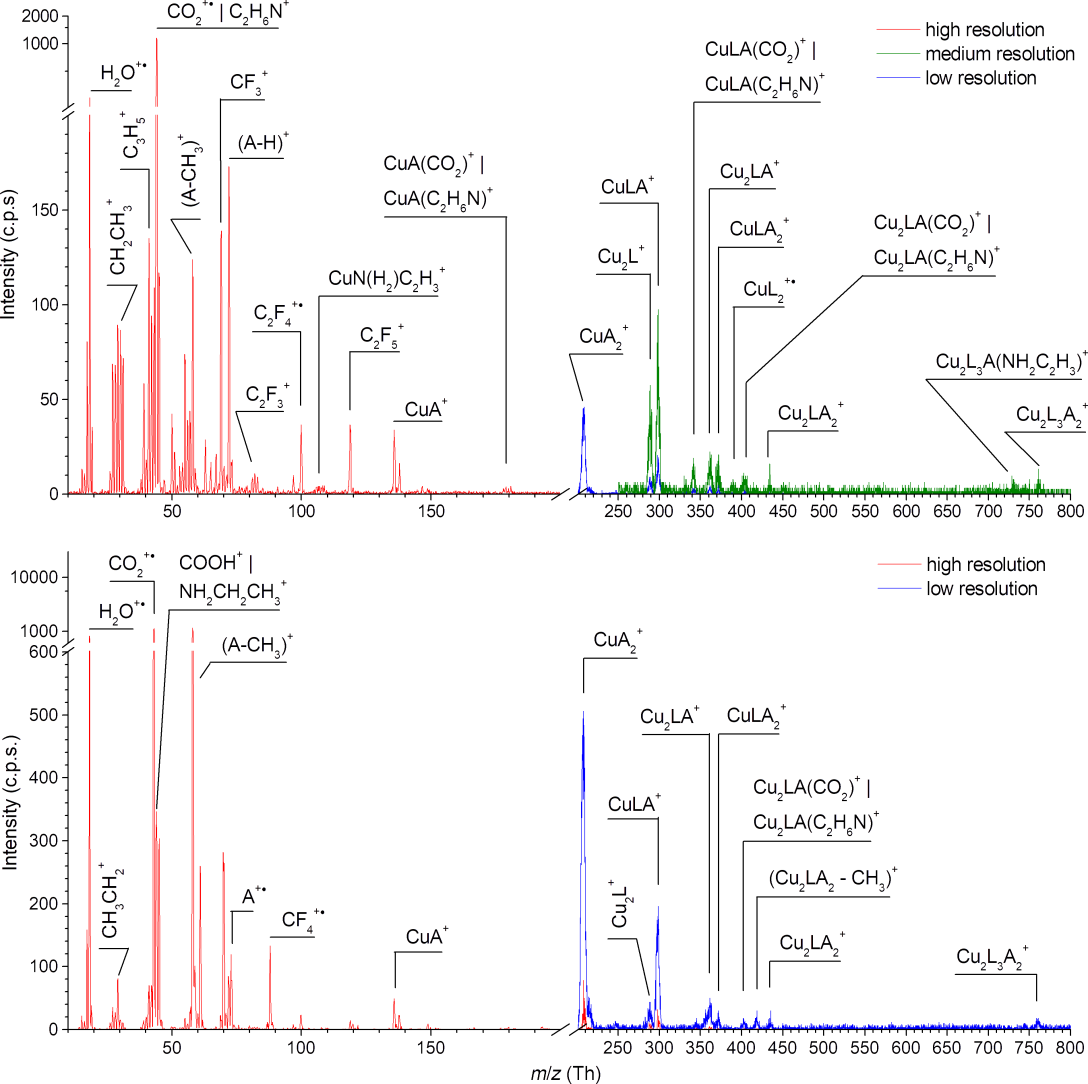
Mass spectrum of [Cu_2_(*s*-BuNH_2_)_2_(µ-O_2_CC_2_F_5_)_4_] molecule (top spectrum) over the range *m*/*z* 10–800 obtained at electron energy 70 eV and mass spectrum of [Cu_2_(*t*-BuNH_2_)_2_(µ-O_2_CC_2_F_5_)_4_] (bottom) obtained at similar conditions (temperature of the beam source of ≈100 °C). For both molecules the spectral range below *m*/*z* 200 was measured with higher mass resolution (red line) but above *m*/*z* 200 (blue line) with low mass resolutions to increase the signal. For [Cu_2_(*s*-BuNH_2_)_2_(µ-O_2_CC_2_F_5_)_4_] molecule (top spectrum) the range above *m*/*z* 250 was also measured with medium resolution (green line).

Similar to the ethylamine complex, the dissociation pattern of both [Cu_2_(*t*-BuNH_2_)_2_(µ-O_2_CC_2_F_5_)_4_] and [Cu_2_(*s*-BuNH_2_)_2_(µ-O_2_CC_2_F_5_)_4_] (Figure 5) is characterized by a lack of parent cation. The highest visible mass (for details Experimental) for both molecules is produced via detachment of one carboxylate ligand (Cu_2_L_3_A_2_^+^). As discussed before for the previous molecules, the range of the ions formed by whole carboxylate or amine ligand loss was registered. They appear for both *tert*-butyl and *sec*-butyl substituents above mass *m*/*z* 130 (Figure 5). We can recognize dicopper fragments, e.g., Cu_2_LA_2_^+^, Cu_2_LA^+^, Cu_2_L^+^ and monocopper ions as follow: CuLA_2_^+^, CuLA^+^, CuA_2_^+^, and CuA^+^. For both complexes the Cu_2_L_2_^+•^ fragment was not detected opposite to the [Cu_2_(µ-O_2_CC_2_F_5_)_4_] and [Cu_2_(EtNH_2_)_2_(µ-O_2_CC_2_F_5_)_4_] spectra. The formation of the CuL_2_^+•^ ion occurred for the [Cu_2_(*s*-BuNH_2_)_2_(µ-O_2_CC_2_F_5_)_4_] only.

The metallated ions formed by the fragmentation of the bonded ligands themselves, e.g., Cu_2_L_3_A(NH_2_C_2_H_3_)^+^, (Cu_2_LA_2_–CH_3_)^+^ filled up mass pattern (Figure 5) but in different ways for the *sec-* and *tert-*butylamine complexes. Moreover, due to the low mass resolution it is not possible to distinguish some fragments, for example the signal at *m*/*z* 405 can be the result of the formation of Cu_2_LA(CO_2_)^+^ or Cu_2_LA(C_2_H_6_N)^+^ fragments.

Fragments with *m*/*z* below 140 are predominantly associated with dissociation and fragmentation of both ligand types. Carboxylate ligand fragments are less abundant for [Cu_2_(*t*-BuNH_2_)_2_(µ-O_2_CC_2_F_5_)_4_]. On the other hand, the fragmentation of the butylamine ligand is dominant for both molecules. Formation of A^+^ ion is not observed for [Cu_2_(*s*-BuNH_2_)_2_(µ-O_2_CC_2_F_5_)_4_], only by stabilization via hydrogen atom detachment and formation of (A–H)^+^. Moreover, formation of fragments with *m*/*z* 44 (C_2_H_6_N^+^ or CO_2_^+•^), and *m*/*z* 58 ((A–CH_3_)^+^) is observed. The *m*/*z* 44 fragment is less dominant for [Cu_2_(*t*-BuNH_2_)_2_(µ-O_2_CC_2_F_5_)_4_] than for the [Cu_2_(*s*-BuNH_2_)_2_(µ-O_2_CC_2_F_5_)_4_] complex. For both molecules we have detected an intensive peak at *m*/*z* 18. Most probably, this is the signal relating to a water impurity as was described above for [Cu_2_(µ-O_2_CC_2_F_5_)_4_]. However, the cation NH_4_^+^ is registered in the pure s-BuNH_2_ and *t*-BuNH_2_ spectra [44] so this possible ion cannot be excluded for the measured compounds at *m*/*z* 18.

Estimates of appearance energies of copper carboxylate complexes with alkylamine ligands have been obtained for both [Cu_2_(EtNH_2_)_2_(µ-O_2_CC_2_F_5_)_4_] (Table 2) and [Cu_2_(*t*-BuNH_2_)_2_(µ-O_2_CC_2_F_5_)_4_] (Table 3). However, the appearance energies were measured only for the fragments with highest intensity due to the charging effects of the monochromator electrodes relating to the measured sample. To avoid the total loss of the signal the electron current was increased which lead to decrease of the electron beam resolution and thus larger uncertainties in the determination of the appearance energies.

**Table 2:** Appearance energies for selected ions from dissociation of [Cu_2_(EtNH_2_)_2_(µ-O_2_CC_2_F_5_)_4_] induced by electron impact ionization.

*m*/*z*	Ion	*AE*^1^[eV]

18	H_2_O^+•^/NH_4_^+^	12.9^a^
30	C_2_H_6_^+•^/CH_2_NH_2_^+^	10.3^a^
44	C_2_H_6_N^+^/CO_2_^+•^	11.4^a^
45	EtNH_2_^+•^	9.0^a^
69	CF_3_^+^	14.1^b^
189	Cu_3_^+^	24.5^d^
270	CuLA^+^	14.6^a^
288	Cu_2_L^+^	12.8^a^
316	CuLA_2_^+^	12.9^a^
334	Cu_2_LA^+^	15.1^b^
704	Cu_2_L_3_A_2_^+^	11.8^c^

^a^Uncertainty ±0.5 eV, ^b^uncertainty ±1 eV, ^c^uncertainty ±2 eV, ^d^uncertainty ±4 eV.

**Table 3:** Appearance energies for selected ions from dissociation of [Cu_2_(*t*-BuNH_2_)_2_(µ-O_2_CC_2_F_5_)_4_] induced by electron impact ionization.

*m*/*z*	Ion	*AE*^1^[eV]	*AE*^2^[eV]

58	(A–CH_3_)^+^	9.1^a^	
119	C_2_F_5_^+^	10.2^a^	15.7^a^
136	CuA^+^	17.0^b^	
193	Cu*t*-Bu_2_NH_2_^+^	15.2^a^	
208	CuA_2_^+^	11.1^a^	15.9^a^
298	CuLA^+^	13.5^b^	

^a^Uncertainty ±0.5 eV, ^b^uncertainty ±1 eV.

**Figure 6:**
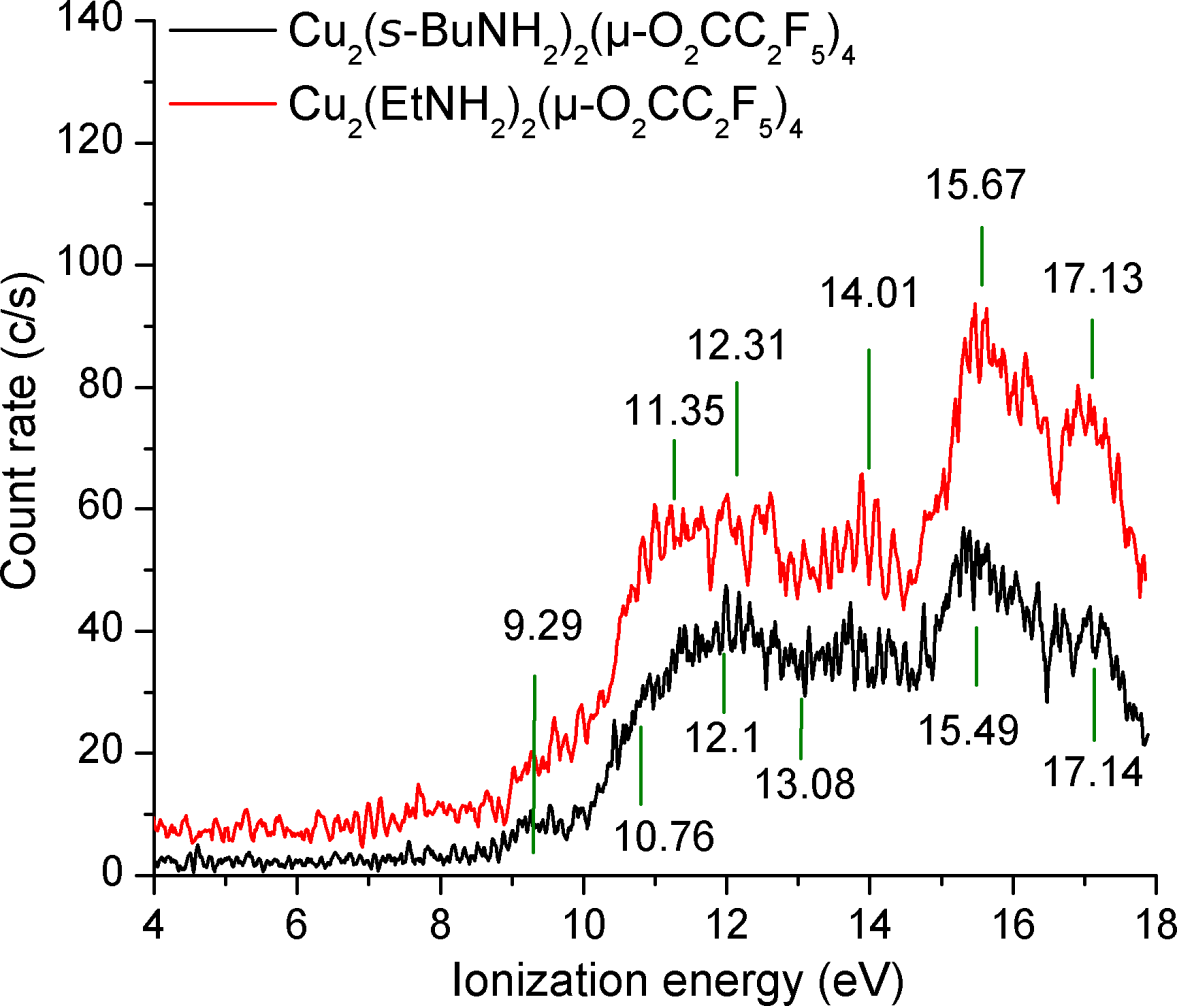
Photoelectron spectra of copper(II) carboxylate complexes [Cu_2_(EtNH_2_)_2_(µ-O_2_CC_2_F_5_)_4_] (red line/upper) and [Cu_2_(*s*-BuNH_2_)_2_(µ-O_2_CC_2_F_5_)_4_] (black line/bottom).

The present results are moreover supported by independent measurements of photoelectron spectra (PES) [45–46] for two of the investigated molecules, namely [Cu_2_(EtNH_2_)_2_(µ-O_2_CC_2_F_5_)_4_] and [Cu_2_(*s*-BuNH_2_)_2_(µ-O_2_CC_2_F_5_)_4_] (Figure 6) while PES intensities were too low for [Cu_2_(*t*-BuNH_2_)_2_(µ-O_2_CC_2_F_5_)_4_]. The obtained data, which are similar for both molecules were interpolated using a set of six gauss functions and energies of the electronic states have been obtained from their maxima. The first electronic transition represents the value of the ionization energy. For both Cu alkylamine complexes the ionization energy was thus estimated to 9.3 eV.

### Negative ions

**Figure 7:**
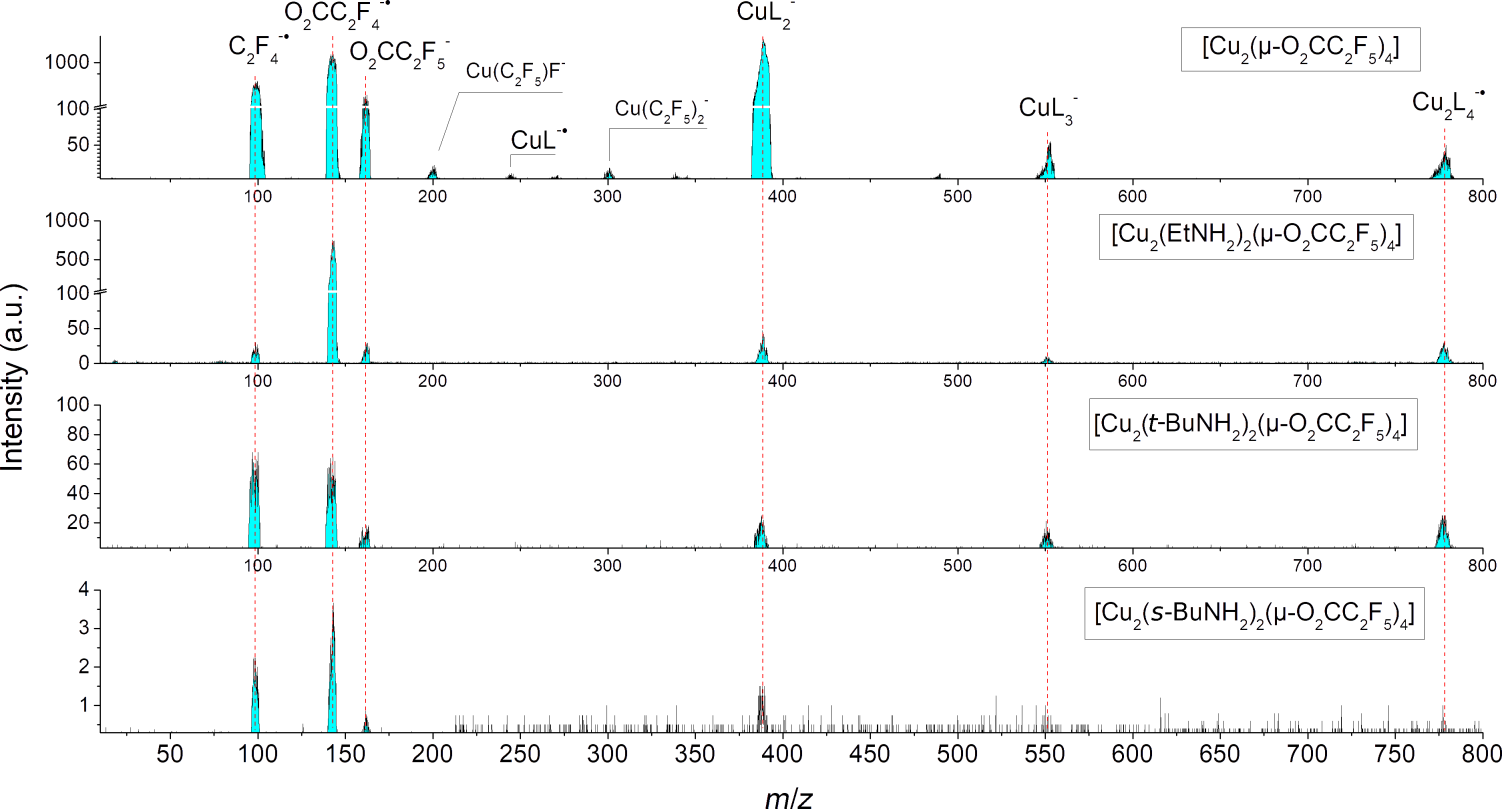
Negative ions mass spectra of copper carboxylate molecules. The spectra were obtained at the energy where maximal ion count of SF_6_^−^ ion production from SF_6_ is observed, which is close to 0 eV.

Mass spectra resulting from electron attachment to all four measured compounds are shown in Figure 7 and reveal many common features. We have observed the production of the transient negative ion (TNI) Cu_2_(O_2_CC_2_F_5_)_4_^−•^ (Cu_2_L_4_^−•^) only for the [Cu_2_(µ-O_2_CC_2_F_5_)_4_] compound. The same Cu_2_L_4_^−•^ ion was detected for both [Cu_2_(EtNH_2_)_2_(µ-O_2_CC_2_F_5_)_4_] and [Cu_2_(*t*-BuNH_2_)_2_(µ-O_2_CC_2_F_5_)_4_] complexes, but with relatively lower ion yields and in this case is the DEA product already and not TNI. The exception was [Cu_2_(*s*-BuNH_2_)_2_(µ-O_2_CC_2_F_5_)_4_], which has the weakest ion yield signal from DEA. Furthermore, DEA leads to production of CuL_3_^−^ and CuL_2_^−^ ions while a weak signal due to CuL^−•^ was detected only for [Cu_2_(µ-O_2_CC_2_F_5_)_4_]. A common feature of DEA to all four measured compounds is that the only dinuclear ionic structure was detected for Cu_2_L_4_^−•^; all the other fragments produced via loss of ligand(s) are associated with one Cu atom only, the dinuclear structure is thus broken.

In addition to copper-containing fragments, the dissociated carboxylate ligand O_2_CC_2_F_5_^−^ was detected. Despite the fact that this structure represents a close shell system, the open shell O_2_CC_2_F_4_^−•^ ion is generally more intense. Moreover, the latter ion can be converted to the C_2_F_4_^−•^ radical anion by loss of carbon dioxide.

**Figure 8:**
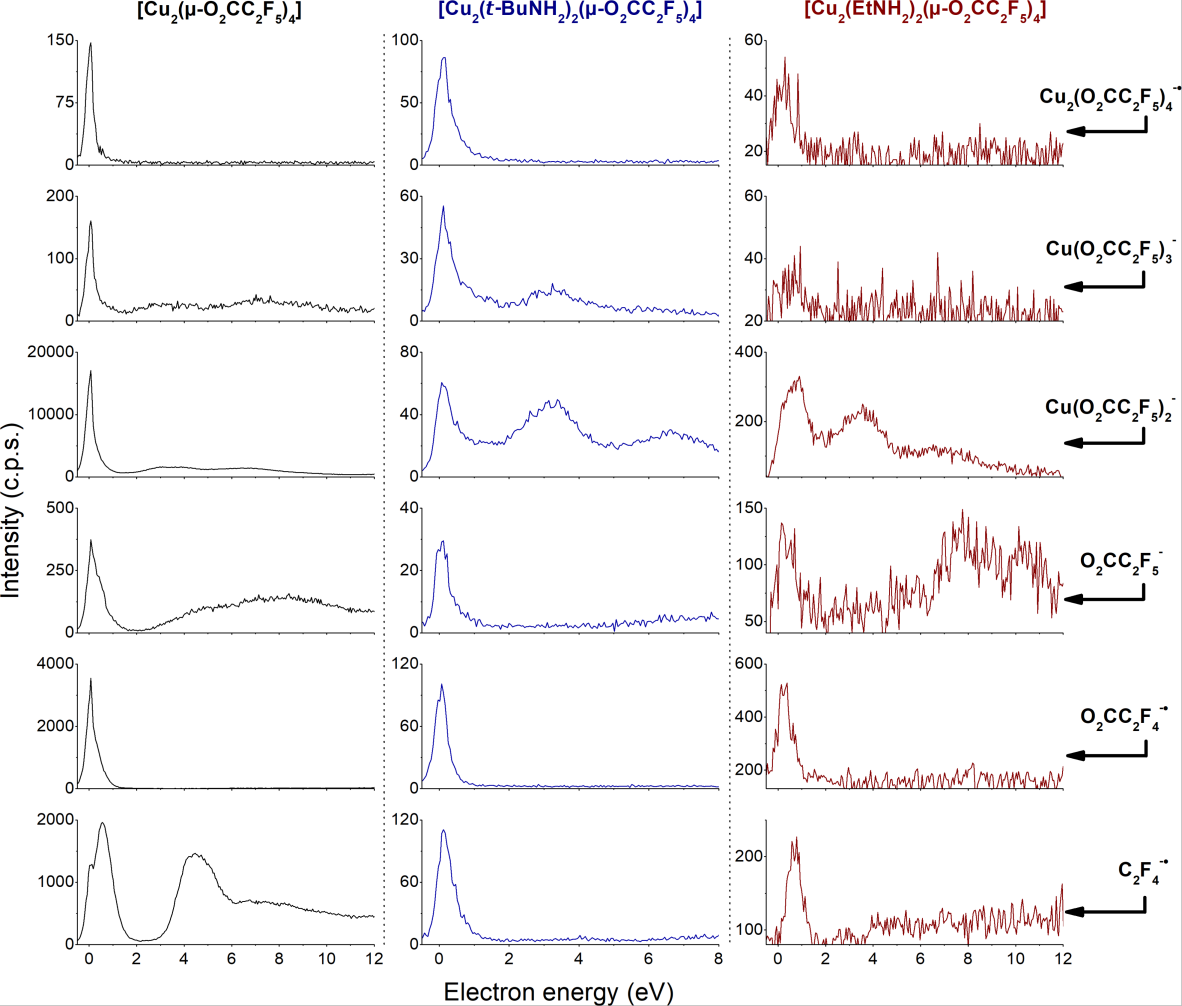
Relative ion yields of negative products from [Cu_2_(µ-O_2_CC_2_F_5_)_4_] (left column), [Cu_2_(*t*-BuNH_2_)_2_(µ-O_2_CC_2_F_5_)_4_] (middle column) and [Cu_2_(EtNH_2_)_2_(µ-O_2_CC_2_F_5_)_4_] (right column) as function of electron energy. Each row represents the negative ion shown on the right. The energy scale was calibrated with respect to the formation of SF_6_^−^ by electron attachment to SF_6_ that occurs at an electron energy ≈0 eV.

Relative DEA cross sections of different negative ions (Cu_2_L_4_^−•^, CuL_3_^−^, CuL_2_^−^, L^−^, O_2_CC_2_F_4_^−•^ and C_2_F_4_^−•^) formed from each of the discussed copper carboxylate complexes were recorded (Figure 8) except for [Cu_2_(*s*-BuNH_2_)_2_(µ-O_2_CC_2_F_5_)_4_] where the signal was insufficient. A strong feature of a single particle resonance close to 0 eV is seen for all DEA products. This is in fact possible only when the electron affinities (EA) of the individual products are large enough to compensate the corresponding bond dissociation energies (BDE) [47]. However, we have no quantitative information about the EA and BDE of the individual products shown in Figure 8. The position of the resonance is slightly shifted towards 1 eV with decreasing the *m*/*z* of the final negative ionic product. This shift is quite obvious for the formation of the C_2_F_4_^−•^ ion as well as for the formation of the Cu(O_2_CC_2_F_5_)_2_^−^ ion from the [Cu_2_(EtNH_2_)_2_(µ-O_2_CC_2_F_5_)_4_] molecule.

## Discussion

### Positive ions

**Figure 9:**
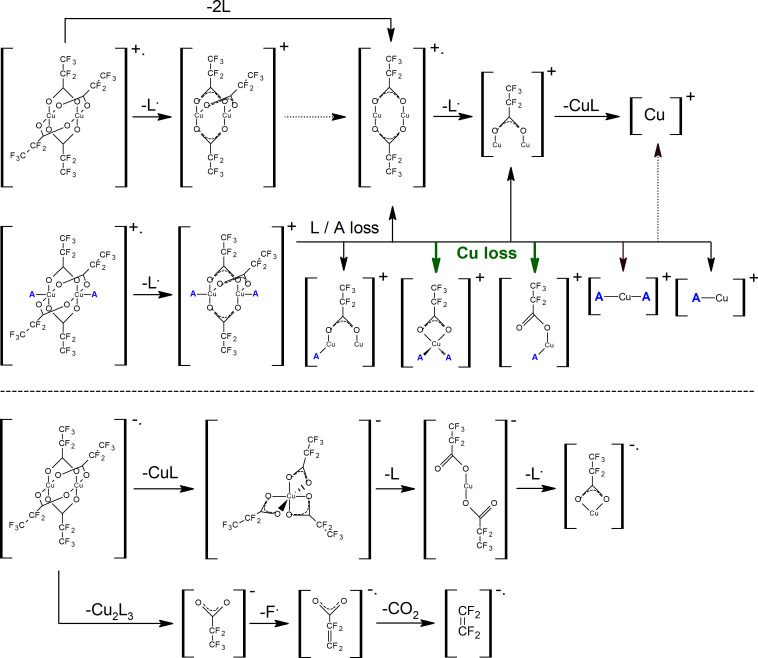
Summary and visualization of the most important ion formation pathways for DI (top) and DEA (bottom).

The dissociation pattern of dissociative ionization of the investigated molecules showed loss of entire ligands as well as fragments containing only copper atom (Figure 9). Limited numbers of fragments containing more than two carboxylate ligands are observed (Cu_2_L_4_^+•^, Cu_2_L_3_^+^, Cu_2_L_3_A_2_^+^). However, it is important to note that intensity of fragments with higher masses is discriminated during their transition through the ion optics and quadrupole system of the experiment.

For [Cu_2_(RNH_2_)_2_(µ-O_2_CC_2_F_5_)_4_] (R = Et, *s*-Bu, *t*-Bu) complexes, the largest detected fragment contained three carboxylate ligands, Cu_2_L_3_A_2_^+^. Moreover, the ions Cu_2_L_3_A^+^ and Cu_2_L_3_A(NH_2_C_2_H_3_)^+^ were formed for R = Et or *s*-Bu, respectively. The presence of the following fragments: Cu_2_L^+^, CuA_2_^+^, CuA^+^ confirms that the copper oxidation state is reduced from two to one. For all three amine complexes similar cationic products containing one or no carboxylate ligand (Cu_2_L_x_A_y_^+^ and CuL_x_A_y_^+^, where x = 0,1; y = 1,2) can be observed. Moreover, for the [Cu_2_(EtNH_2_)_2_(µ-O_2_CC_2_F_5_)_4_] amine complex, the production of Cu^+^ atomic ion is visible, but negligible in contrast to the [Cu_2_(µ-O_2_CC_2_F_5_)_4_] molecule. The formation of the Cu_2_^+•^ ion was not observed as well.

Appearance energies could be measured for almost thirty different fragments of the studied molecules, (excluding [Cu_2_(*s*-BuNH_2_)_2_(µ-O_2_CC_2_F_5_)_4_]), but not for the intact complex. As ionization energies were thus not obtained for any of the measured molecules by the electron–molecular beam experiment, PES were acquired for [Cu_2_(EtNH_2_)_2_(µ-O_2_CC_2_F_5_)_4_] and [Cu_2_(*s*-BuNH_2_)_2_(µ-O_2_CC_2_F_5_)_4_] yielding ionization energies of *IE* = 9.3 eV. Based on the very similar structure, a similar *IE* is expected for [Cu_2_(*t*-BuNH_2_)_2_(µ-O_2_CC_2_F_5_)_4_] while the ionization energy of [Cu_2_(µ-O_2_CC_2_F_5_)_4_] may be markedly different due to the absence of alkylamine ligands.

As shown in Figure 3 and listed in Table 1, we have obtained for [Cu_2_(µ-O_2_CC_2_F_5_)_4_] appearance energies for sequential loss of carboxylate ligands. Usually, the dissociation of ligands from coordination compounds requires higher incident electron energies with increasing number of ligands dissociated from the complex [17–19]. However, here the loss of two carboxylate ligands is energetically the lowest with *AE*_452_ = 9.7 eV. Dissociation of first and third carboxylate ligands occur with similar values of *AE_615_* = 11.4 eV and *AE*_289_ = 11.9 eV, respectively. Thus dissociation does not follow a simple one by one ligand loss mechanism. The similar values of *AE*_615_ and *AE*_289_ may be explained only in the case, that while the formation of Cu_2_(O_2_CC_2_F_5_)_3_^+^ ion occur as a dissociation process from the parent cation, the second threshold at 15.2 eV for formation of Cu_2_(O_2_CC_2_F_5_)_2_^+^ would represent the spontaneous dissociation of the carboxylate ligand from the Cu_2_(O_2_CC_2_F_5_)_3_^+^ ion. The formation of Cu_2_(O_2_CC_2_F_5_)^+^ is a result of ligand loss from Cu_2_(O_2_CC_2_F_5_)_2_^+•^. We can thus estimate the bond dissociation energy (as the difference of appearance energies of the corresponding ions) *BDE* [Cu_2_(O_2_CC_2_F_5_)_2_^+•^–(O_2_CC_2_F_5_)] = *AE*_452_ − *AE*_615_ = 3.8 eV, *BDE* [Cu_2_(O_2_CC_2_F_5_)^+^–(O_2_CC_2_F_5_)] = *AE*_289_ − *AE*_452_ = 2.3 eV.

The measured appearance energies of CuLA_2_^+^ (*AE*_316_ = 12.9 eV) and CuLA^+^ (*AE*_270_ = 14.6 eV) produced from [Cu_2_(EtNH_2_)_2_(µ-O_2_CC_2_F_5_)_4_] show that *BDE* [Cu(EtNH_2_)(O_2_CC_2_F_5_)^+^–EtNH_2_] ≈ 1.7 eV. On the other hand the comparison of *AE*s of the CuLA^+^ with its dinuclear counterpart Cu_2_LA^+^ prefers in energy the formation of the smaller fragment CuLA^+^ (*AE*_270_ = 14.6 eV) contrary to Cu_2_LA^+^ (*AE*_334_ = 15.1 eV).

The dissociation pattern of the carboxylate ligand is visible well for the [Cu_2_(µ-O_2_CC_2_F_5_)_4_] molecule. Its fragmentation can be compared with the dissociation pattern of a similar molecule, the pentafluoropropionic acid C_2_F_5_COOH [48]. Similarly as for the C_2_F_5_COOH we have seen formation of C_2_F_5_^+^ with *m*/*z* 119, C_2_F_4_^+•^ with *m*/*z* 100, CF_3_^+^ with *m*/*z* 69 or CF^+^ with *m*/*z* 31. We can clearly observe that the C_2_F_5_^+^ formation (*AE*_119_ = 15.1 eV) requires more energy than dissociation of one additional fluorine atom to form the C_2_F_4_^+•^ ion (*AE*_100_ = 12.2 eV). The dissociation of a fluorine atom can lead to a C=C double bond in C_2_F_4_^+^ which in fact compensates the energy needed for the dissociation of the C–F bond and can be observed as a energy decrease of *AE*_100_ in comparison to *AE*_119_. The formation of CF_3_^+^ ion with *m*/*z* 69 is detected with two thresholds at *AE*^1^_69_ = 11.7 eV and *AE*^2^_69_ = 16.1 eV. Loss of one more fluorine atom leads to CF_2_^+^ (*m*/*z* 50) with *AE*_50_ = 14.4 eV. The formation of CF^+^ ion with *m*/*z* 31 is again detected with two thresholds at *AE*^1^_31_ = 11.0 eV and *AE*^2^_31_ = 18.4 eV. The first threshold occurs at the energy, which is below the dissociation limit for CF_3_ as well as CF_2_ ions. From the dissociation sequence we can estimate the bond dissociation energy of the fluorine atom in the present CF_3_^+^ ion as: *BDE* [CF_2_^+^–F] = 2.7 eV and *BDE* [CF^+^–F] = 4.0 eV. For comparison, the same energies for the CF_4_ molecule after EI are *BDE* [CF_2_^+^–F] ≈6 eV and *BDE* [CF^+^–F] = 2.3 eV [49–50]. An additional comparison may be provided with hexafluoroethane (C_2_F_6_) [51], where the appearance energy difference between CF_3_^+^ and CF^+^ ion is ≈3 eV in relation to almost 7 eV difference for the [Cu_2_(µ-O_2_CC_2_F_5_)_4_] compound. Moreover, the formation of the C_2_F_4_^+•^ ion requires 5.7 eV more than formation of C_2_F_5_^+^ ion from hexafluoroethane. We detected an opposite trend with the difference of slightly over 3 eV. This significant effect is provided by the presence of the carboxyl group.

For all three studied complexes, the fragmentation of the alkylamine ligand cation is observed with higher intensity than that of the carboxylate ligand. For [Cu_2_(EtNH_2_)_2_(µ-O_2_CC_2_F_5_)_4_] and [Cu_2_(*s*-BuNH_2_)_2_(µ-O_2_CC_2_F_5_)_4_], it is produced as a deprotonated (A–H)^+^ ion instead of A^+^, visible in the spectrum of [Cu_2_(*t*-BuNH_2_)_2_(µ-O_2_CC_2_F_5_)_4_]. In the case of [Cu_2_(EtNH_2_)_2_(µ-O_2_CC_2_F_5_)_4_], the fragmentation leads only to the formation of a fragment with *m*/*z* 30 as assigned to C_2_H_6_^+•^ or more probable to H_2_NCH_2_^+^. The registered spectrum is the superposition of the thermal loss EtNH_2_ and the complex spectra what influenced the observed signals intensities. Mass spectra for *sec*-butylamine and *tert*-butylamine show one dominant dissociation product: H_2_NC(H)CH_3_^+^ with *m*/*z* 44 and H_2_NC(CH_3_)_2_^+^ with *m*/*z* 58, respectively [44]. Both of these processes are also observed in corresponding dissociation patterns of the molecules investigated here. The ion with *m*/*z* 44 may also be associated with CO_2_^+•^. However, the appearance energy of *m*/*z* 44 from [Cu_2_(EtNH_2_)_2_(µ-O_2_CC_2_F_5_)_4_] is lower than that of CO_2_^+•^ from CO_2_ [52]. This points to an assignment to (A–H)^+^ as may also be expected for the s-BuNH_2_ and *t*-BuNH_2_ complexes. Moreover, the *AE* detected for H_2_NC(CH_3_)_2_^+^ (*m*/*z* 58) from [Cu_2_(*t*-BuNH_2_)_2_(µ-O_2_CC_2_F_5_)_4_] of *AE*_58_ = 9.1 eV is similar to the PES value of its *IE =* 9.3 eV. We can thus conclude that the dissociation of the amine ligand from the complex and the consequential dissociation of one methyl group as well as the second methyl group are extremely effective processes. For [Cu_2_(*s*-BuNH_2_)_2_(µ-O_2_CC_2_F_5_)_4_], the loss of an ethyl group is a dominant product of alkylamine ligand fragmentation. Additionally, the single methyl group dissociation is observed. Other remaining fragments of the studied molecules relate to additional hydrogen or carbon dissociations.

### Negative ions

In the fragmentation pattern obtained via DEA, we observed with particularly high intensity the symmetrical splitting of the Cu_2_(O_2_CC_2_F_5_)_4_^−•^ ion into the Cu(O_2_CC_2_F_5_)_2_^−^ and Cu(O_2_CC_2_F_5_)_2_ fragments. Therefore, we can suppose that the lowest unoccupied molecular orbital (LUMO) of the measured complexes has an antibonding character and consists from d-orbitals on Cu atoms, and p-orbitals from the corresponding O atoms. However, Figure 7 shows a difference in the relative intensity of Cu(O_2_CC_2_F_5_)_2_^−^ ion formed via DEA for all four compounds. While it is the most intensive product for the [Cu_2_(O_2_CC_2_F_5_)_4_] measured at electron energy close to 0 eV, its intensity is significantly lower in the spectra of the other three complexes, measured at the same electron energy. The maximum of this DEA channel in the [Cu_2_(RNH_2_)_2_(µ-O_2_CC_2_F_5_)_4_] compounds is shifted towards higher energies (Figure 8), which in fact is a consequence of more bonds to be dissociated to produce the same product Cu(O_2_CC_2_F_5_)_2_^−^. Moreover, the Cu–N bond is involved in the DEA, as well as one of the Cu–O bonds of each Cu-carboxyl part of the complex. Thus, DEA to the all complexes leading to Cu_2_(O_2_CC_2_F_5_)_4_^−•^ was observed. At the same point if it is the antibonding character of Cu–O bonds and interactions between copper ions then it gives rise to ionic products Cu(O_2_CC_2_F_5_)_3_^−^, Cu(O_2_CC_2_F_5_)_2_^−^, and Cu(O_2_CC_2_F_5_)^−•^. The ion yield for these reactions peaks close to 0 eV. Fragmentations with additional ligand dissociation processes have been observed, forming still well visible peaks with *m*/*z* ≈201 and *m*/*z* ≈301. The signals can be assigned to Cu(C_2_F_5_)F^−^ for the peak with *m*/*z* ≈201 and Cu(C_2_F_5_)_2_^−^ for the peak with *m*/*z* ≈301. For [Cu_2_(µ-O_2_CC_2_F_5_)_4_], the dissociation through higher energy resonances leads only to smaller fragments (Figure 8). [Cu_2_(EtNH_2_)_2_(µ-O_2_CC_2_F_5_)_4_] and [Cu_2_(*t*-BuNH_2_)_2_(µ-O_2_CC_2_F_5_)_4_], similar to [Cu_2_(µ-O_2_CC_2_F_5_)_4_], are characterized by the same DEA products. Moreover, new energetic channels leading to Cu(O_2_CC_2_F_5_)_2_^−^ areas appear at 3.6 eV and 6.4 eV for [Cu_2_(*t*-BuNH_2_)_2_(µ-O_2_CC_2_F_5_)_4_] and at 3.7 eV and 7 eV for [Cu_2_(EtNH_2_)_2_(µ-O_2_CC_2_F_5_)_4_]. The experimental results show that DEA to these complexes will also lead to formation of the ligand O_2_CC_2_F_5_^−^ anion. This ion exists as a stable non-radical anion, but its further dissociation has been detected. The presence of negative charge can affect the central carbon of the pentafluoropropionate, which leads to a loss of a fluorine atom. In fact, the O_2_CC_2_F_4_^−•^ anion represents the most abundant product among the ions from ligand dissociation. Finally, we have observed the formation of C_2_F_5_^−^ as the result of the carbon dioxide dissociation from O_2_CC_2_F_5_^−^. In addition a slight shift of resonance maxima can be observed, which can represent a second resonance and/or a significant contribution of higher vibrational modes for an effective dissociation. In the case of [Cu_2_(µ-O_2_CC_2_F_5_)_4_], the contribution of higher excited states is significant, especially for a resonance at an incident electron energy ≈4.4 eV. This higher energy resonance could have its origin on a pentafluoroethyl substituent for [Cu_2_(µ-O_2_CC_2_F_5_)_4_] exclusively leading to the dissociation of the C_2_F_5_^−^ ion directly from TNI. The position of the resonance agrees with the formation of C_2_F_5_^−^ anion from C_2_F_6_ molecule, its maximum cross section is here located at a similar energy of 4.8 eV [53]. The presence of EtNH_2_ or *t*-BuNH_2_ in the complex closes this channel and the C_2_F_5_^−^ anion is formed only via the single particle resonance at almost 0 eV.

## Conclusion

This article presents an investigation of the fragmentation following electron impact ionization of and electron attachment to four copper(II) carboxylate complexes.

Regarding electron impact ionization, the cross sections for formation of the parent molecular ions were very weak. Therefore, PES experiment have been performed for [Cu_2_(EtNH_2_)_2_(µ-O_2_CC_2_F_5_)_4_] and [Cu_2_(*s*-BuNH_2_)_2_(µ-O_2_CC_2_F_5_)_4_] to determine the ionization energies as 9.3 eV for both compounds. Appearance energies show lower thresholds for loss of a ligand pair as compared to for loss of single ligands. This effect is quite obvious for [Cu_2_(µ-O_2_CC_2_F_5_)_4_], where *AE*(Cu_2_(O_2_CC_2_F_5_)_3_^+^) = 11.4 eV, *AE*(Cu_2_(O_2_CC_2_F_5_)_2_^+•^) = 9.7 eV, and *AE*(Cu_2_(O_2_CC_2_F_5_)^+^) = 11.7 eV. The fragmentation of the ligand is also observed and comparable with respect to the suitable amine or carboxylic acid. The observation of the metallated ions containing both ligand types, e.g., [Cu_2_(O_2_CC_2_F_5_)(RNH_2_)]^+^, [Cu_2_(O_2_CC_2_F_5_)_3_(RNH_2_)_2_]^+^ confirmed whole complex molecules evaporation. However, in the case of the [Cu_2_(EtNH_2_)_2_(µ-O_2_CC_2_F_5_)_4_] complex a partial amine loss occurred. The substantial production of free Cu^+^ was detected only for [Cu_2_(µ-O_2_CC_2_F_5_)_4_] molecule, and a very weak production for [Cu_2_(EtNH_2_)_2_(µ-O_2_CC_2_F_5_)_4_] complex. For the remaining two complexes we did not detect any Cu^+^ ions. Therefore, we conclude that [Cu_2_(µ-O_2_CC_2_F_5_)_4_] can efficiently decompose to Cu^+^ ion via electron impact in FEBID. The amine ligand introduction decreased the evaporation temperature but unfortunately suppressed the copper ion formation. On the other hand, this phenomenon can be useful for the “halo” effect limitation in FEBID processes.

Regarding electron attachment, we have registered the first spectra of negative ions for copper carboxylates compounds. Comparable negative ions are formed for all investigated molecules. The electron attachment processes occur mainly at incident electron energy close to 0 eV, through single particle resonances but specific fragments are also formed with smaller intensity through higher-lying resonances. In all cases (for [Cu_2_(*s*-BuNH_2_)_2_(µ-O_2_CC_2_F_5_)_4_] only a week signal was observed), dissociative electron attachment generates the Cu_2_(O_2_CC_2_F_5_)_4_^−•^ anion. Dissociation causes the splitting of molecules into two almost equal fragments and thus formation of the Cu(O_2_CC_2_F_5_)_2_^−^ anion. This ion is dominant in the case of [Cu_2_(µ-O_2_CC_2_F_5_)_4_] but not as pronounced when the amine is coordinated. The formation of the negative ion of the carboxylate ligand O_2_CC_2_F_5_^−^ was detected together with additional dissociation fragments O_2_CC_2_F_4_^−•^ and C_2_F_4_^−•^. Here, the generation of the O_2_CC_2_F_4_^−•^ anion represents the very stable and abundant structure.

## Experimental

Investigation of electron induced processes was carried out by crossed electron and molecular beam experiments [54]. The electron beam was created by a trochoidal electron monochromator operating with energy resolution down to 100 meV in the range 0–120 eV. In the case of low signals, which were either an inherent property of the sample or resulted from the effect of deposition of the sample on the monochromators electrodes, the electron resolution was reduced up to 300–500 meV. A molecular beam was created by sublimation/evaporation of solid/gel samples into a small chamber. The chamber is connected with a main reaction chamber by small capillary, which creates a molecular beam that perpendicularly collides with the electron beam. Ionic products are then forced by a weak electric field into the ion optics of the quadrupole mass spectrometer. After separation of the products with different mass-to-charge ratios (*m*/*z*) the ions are detected by an electron multiplier. A constant electron energy of 70 eV was applied to register the mass spectra, i.e., the ion intensity as function of the *m*/*z* ratio of the measured ions. For a selected product (selected *m*/*z* ratio) the ion yield dependences were then measured by varying the electron energy. In the case of the negative ions the recorded mass spectrum strongly depends on the electron energy due to a resonant character of attachment reaction.

For the measured cross section of electron ionization and dissociative ionization we can evaluate the threshold value of the corresponding ion formation by a fitting procedure using a modified Wannier law [55]. This value then represents an ionization potential or appearance energy of electron ionization or dissociative ionization respectively.


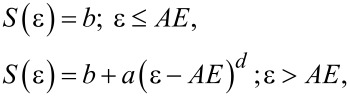


where *b* represent background, *AE* represent appearance (or ionization) energy, ε is electron energy and *a, d* are independent fitting parameters.

Calibration of the electron energy has been carried out by measurement of the ionization potential of Ar atoms and calibration to its known value 15.76 eV [56] and with reference to the maximum of the electron attachment resonance on SF_6_ molecule at energy ≈0 eV [57].

The studied complexes were heated up to temperature of 80–100 °C, except for [Cu_2_(µ-O_2_CC_2_F_5_)_4_] where the temperature range was 140–160 °C. Investigated molecules are characterized by relative high masses in range of 778 amu for [Cu_2_(µ-O_2_CC_2_F_5_)_4_] up to 924 amu for [Cu_2_(*t*-BuNH_2_)_2_(µ-O_2_CC_2_F_5_)_4_] or [Cu_2_(*s*-BuNH_2_)_2_(µ-O_2_CC_2_F_5_)_4_]. In the present experiment the intensity of ions detected with masses above approximately *m*/*z* 300 is reduced by the QMS. This can be avoided by decreasing the mass resolution (usually defined as the ratio of mass *m* and full width at half maximum of the peak Δ*m*) through changing the software parameter, which defines the resolution of the ion peak in the mass spectrum (see, for instance, Figure 2). Heavy ions can thus be detected. However, the position of the peak is then not measured precisely as the signal is broadened over several masses. High resolution measurements presented in the paper represent a peak FWHM ≈0.6 amu and for medium resolution ≈1.6 amu. Measurements with low and very low resolution yield FWHM of peaks ≈4.5 both, however with higher transmittance for the second one. (Regular *m*/Δ*m* ratio can be hardly evaluated due to dynamic resolution behavior.)

Photoelectron spectra (PES) were registered with a Perkin Elmer He I photoelectron spectrometer [45–46]. Photons with energy 22.21 eV ionize the studied molecules in the gas phase. The photoelectrons depart from the chamber through a narrow slit and are analyzed with a cylindrical electrostatic analyzer. In the present measurements, electrons pass through an analyzer at the fixed predefined energy, while the potential of the analyzer is varied with respect to the target chamber. An energy calibration was carried out by measuring known argon and xenon ionization potentials.

### Materials

Copper(II) carboxylate compounds with *tert*-butylamine of the general formula [Cu_2_(*t*-BuNH_2_)_2_(µ-O_2_CR)_4_], where R = C*_n_*F_2_*_n_*_+1_, *n* = 1–6, were obtained in the reaction of copper(II) perfluorinated carboxylates with *tert*-butylamine, which was in situ generated from *tert*-butyl isocyanate [34]:


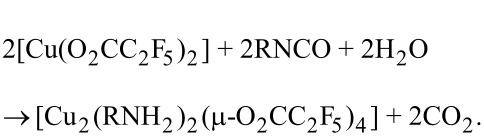


The analogues procedure was applied for the synthesis of new complexes [Cu_2_(EtNH_2_)_2_(μ-O_2_CC_2_F_5_)_4_] and [Cu_2_(*s*-BuNH_2_)_2_(μ-O_2_CC_2_F_5_)_4_].

Copper(II) carboxylate [Cu(O_2_CC_2_F_5_)_2_] was prepared as reported [58]. Ethyl isocyanate (98%), *sec*-butyl isocyanate (s-BuNCO, 98%), and acetonitrile (99.93%) were purchased from Aldrich. All reagents were used as received.

### Instrumentation for complexes characteristics

The first mass spectra were detected with a Finnigan MAT 95 mass spectrometer, using electron ionization (EI) method over the temperature range 30–350 °C. IR spectra were measured with a PerkinElmer Spectrum 2000 FTIR spectrometer and a Spectrum RXI PerkinElmer, using KBr plates (400–4000 cm^−1^). The Cu content was determined with a Varian Spectr AA-20 Plus spectrophotometer. The content of C and H was determined CHNS Elemental Analyser-Euro Vector model 3018.

The yield of the complexes synthesis was about 60%. The results of elementary analyses and spectroscopic data for new compounds were following:

**[Cu_2_(EtNH_2_)_2_(µ-O_2_CC_2_F_5_)_4_]** C_16_H_14_Cu_2_F_20_N_2_O_8_ (calc./found) % Cu 14.6/14.2, C 22.1/22.1, H 1.63/2.41, EIMS *T* = 58 °C (*m*/*z*, RI %) C_2_H_7_N^+•^ (45, 5); Cu_2_(O_2_CC_2_F_5_)^+^ (289, 100); Cu_2_(EtNH_2_)(O_2_CC_2_F_5_)^+^ (334, 3); Cu_2_(O_2_CC_2_F_5_)_2_^+^ (452, 40); Cu_2_(EtNH_2_)_2_(O_2_CC_2_F_5_)_3_^+^ (705, 4), IR (KBr): , 3244, 3075, 2993, 2835, 2738, 2624, 2528, 2087, 1675, 1531, 1479, 1462, 1413, 1326, 1212, 1161, 1030, 821, 799, 733, 585, 541, 422 cm^−1^.

**[Cu_2_(s-BuNH_2_)_2_(µ-O_2_CC_2_F_5_)_4_]** C_20_H_22_Cu_2_F_20_N_2_O_8_ (calc./found) % Cu 13.7/14.2, C 26.0/25.4, N 2.4/3.8, H 2.4/3.7, EIMS *T* = 145 °C (*m*/*z*, RI %) C_2_H_5_N^+^/CO_2_^+•^ 44, 100; C_3_H_8_N^+^ 58, 14; C_2_F_4_^+•^ 100, 41; C_2_F_5_^+^ 119, 32; Cu(s-BuNH_2_)^+^ 136, 2; Cu_2_F^+^ 145,5; Cu_2_(O_2_CC_2_F_5_)^+^ 289, 33; Cu_2_(O_2_CC_2_F_5_)_2_^+•^ 452, 13; Cu_2_(O_2_CC_2_F_5_)_3_(*s*-BuNH_2_)_2_^+^ 761, 2; IR (KBr): 3240, 3121, 2980, 2945, 2892, 2747, 2655, 2567, 1678, 1612, 1518, 1489, 1465, 1419, 1327, 1213, 1164, 1033, 823, 775, 733, 586, 540, 483, 448 cm^−1^.
